# Serum creatinine to cystatin C ratio as a biomarker for monitoring motor-function in children with spinal muscular atrophy treated with nusinersen: a retrospective cohort study

**DOI:** 10.1186/s12883-026-04657-3

**Published:** 2026-01-24

**Authors:** Yuan yuan Zhang, Jie Wang, Zhen qiong Cui, Kai Ma

**Affiliations:** 1 Department of Paediatrics, Affiliated Liaocheng Second People’s Hospital of Shandong First Medical University, Liaocheng, Shandong Province China; 2 Department of Neurology, Jinan Children’s Hospital (Children’s Hospital Affiliated to Shandong University), Jinan, Shandong Province China

**Keywords:** Spinal muscular atrophy, Serum biomarkers, Nusinersen, Creatinine-to-cystatin C ratio, Children, Retrospective cohort study

## Abstract

**Objective:**

To validate the clinical utility of creatinine-to-cystatin C ratio (CCR) as a biomarker for monitoring nusinersen treatment response in Chinese paediatric patients receiving nusinersen monotherapy.

**Methods:**

In this retrospective, single-center study, 33 genetically confirmed 5q-SMA patients (≤ 18 years) treated with intrathecal nusinersen for ≥ 26 months (2020.2–2025.6) were enrolled. Motor function was serially evaluated using the Hammersmith Functional Motor Scale Expanded (HFMSE), Revised Upper Limb Module (RULM), and Hammersmith Infant Neurological Exam Part 2 (HINE-2). Serum biomarkers, such as creatinine (Cr), creatine kinase (CK), and cystatin C, were measured at multiple time points. A linear mixed-effects model (adjusted for age, body mass index, SMA type, and treatment duration) was used to assess the biomarker–function associations.

**Results:**

Ambulant patients and those with four SMN2 copies demonstrated elevated CCR and Cr levels compared to non-ambulant patients and those with three SMN2 copies at baseline. Serum CCR levels were significantly higher than the baseline at V4–V8 (10-26 months). Cr levels were significantly higher than the baseline at V8 (26 months), whereas CK and cystatin C levels remained unchanged. In the fully adjusted linear mixed-effects models, both CCR and Cr were positively associated with HFMSE and RULM scores. However, only CCR remained significant after adjustment. Cystatin C levels were inversely correlated with the HINE-2 scores.

**Conclusion:**

CCR is a promising biomarker in paediatric SMA patients receiving nusinersen monotherapy; however, its validity and generalizability require confirmation in larger, more diverse multicenter cohorts.

**Supplementary Information:**

The online version contains supplementary material available at 10.1186/s12883-026-04657-3.

## Introduction

 Spinal Muscular Atrophy (SMA) is an autosomal recessive disorder with devastating consequences, primarily originating from survival motor neuron (SMN) 1 gene homozygous deletions/mutations (5q11.2-q13.3). This condition triggers the progressive degeneration of spinal α-motor neurons, culminating in skeletal muscle atrophy and weakness [1]. Globally, SMA is a predominant genetic contributor to infant death, with a birth prevalence of approximately 1 in 6, 000–10, 000 and carrier rates of 1:40–60 [2, 3]. It imposes substantial and enduring burdens on affected children, families, and healthcare infrastructure throughout their lives. Phenotypic manifestations range from fatal infantile-onset forms (types 0/1, necessitating ventilation) to attenuated later-onset variants (types 2–4). All subtypes share core features, including progressive motor deterioration, respiratory dysfunction, and severe functional deficits [4].

Disease-modifying therapies (DMTs), particularly the antisense oligonucleotide nusinersen (Spinraza^®^), which boosts functional SMN protein synthesis via SMN2, have reshaped SMA therapeutic approaches [5, 6]. Nusinersen shows significant therapeutic benefits in motor function stabilization or enhancement across disease subtypes, especially when administered pre-symptomatically or early [7–9]. Accurate monitoring of treatment responses requires reliable biomarkers that augment clinical evaluations [10]. Established functional assessments, such as the Hammersmith Functional Motor Scale Expanded (HFMSE), Revised Upper Limb Module (RULM), and Hammersmith Infant Neurological Exam-Part 2 (HINE-2), provide valuable information. However, they require specialized training and considerable time, display floor/ceiling limitations, and often fail to detect minor alterations, notably among non-ambulatory individuals [10–14]. Therefore, there is a need for reliable biomarkers to complement the clinical evaluation.

Serum biomarkers offer a promising alternative for tracking disease progression and treatment response in a more objective, quantifiable, and minimally invasive manner. Creatine kinase (CK), a standard indicator of muscular injury, displays inconsistent relationships with disease severity and treatment outcomes in adult SMA [15], while paediatric evidence remains scarce [10]. Serum creatinine (Cr), representing the terminal product of skeletal muscle creatine metabolism, functions as a candidate biomarker; diminished Cr levels are linked to increased severity, reduced SMN2 copy numbers, and loss of ambulation in adolescents/adults [16–19]. Nusinersen treatment correlates with elevated serum Cr concentrations and enhanced motor performance scores [17, 18], implying its clinical monitoring value. Nevertheless, Cr interpretation is complicated by renal parameters (especially glomerular filtration rate, GFR), muscle mass, and nutritional protein intake, which constrain its dependability in children [19].

Cystatin C (CysC), a cysteine protease inhibitor unaltered by muscular metabolism or demographic variables, is a sensitive endogenous indicator of GFR [20]. To address the limitations of Cr, the creatinine-to-cystatin C ratio (CCR) has been developed as a mechanistically refined surrogate marker for muscle mass evaluation. CCR counteracts GFR interference and establishes significant associations with skeletal muscle volume, strength capacity, and functional performance [21–24], including the diagnostic value for sarcopenia and mortality forecasting [23]. Importantly, among nusinersen-treated adults, CCR showed dynamic correlations with HFMSE measurements and increased substantially from baseline [14]. However, its ability to monitor muscular changes and therapy responses in paediatric SMA, where renal maturation and muscle development are ongoing, remains unproven.

Given these considerations, this study aimed to assess the validity of CCR, alongside CK, Cr, and CysC, as biomarkers for monitoring motor function in paediatric SMA patients treated with nusinersen over a 26-month period. We postulated that serum CCR, owing to its enhanced muscle mass representation, would display significant positive associations with standardised motor assessments (HFMSE, RULM, and HINE-2) and detect nusinersen-mediated changes. Validating CCR as a dependable biomarker could refine precision in tracking SMA progression and therapeutic effects, enabling individualized management approaches and boosting clinical trial efficacy via objective physiological linkages to functional endpoints.

## Methods

### Study design and setting

This retrospective single-institution cohort analysis was performed at Jinan Children’s Hospital (February 2020-June 2025). Consecutive paediatric cases fulfilling two criteria were enrolled: (i) molecularly verified 5q-SMA diagnosis and (ii) full intrathecally administered nusinersen induction-maintenance protocol (initial doses: days 0, 14, 28, 63; subsequent 4-monthly) plus ≥ 26 months post-baseline monitoring. Diagnostic confirmation necessitated either homozygous SMN1 deletion or compound heterozygous SMN1 deletion harboring pathogenic variants, supported by concordant clinical manifestations.

### Participants

The inclusion criteria were as follows: 1) genetically confirmed age < 18 years receiving nusinersen monotherapy and 2) comprehensive baseline and serial laboratory/functional evaluations over 26 months of follow-up. The exclusion criteria were as follows: i) nusinersen commencement preceding/following the study timeframe, ii) co-administration or prior use of alternative SMN-directed therapeutics (onasemnogene abeparvovec/risdiplam), and iii) essential covariate incompleteness.

### Biomarker measurements

Peripheral blood collection synchronized with nusinersen delivery at eight intervals: baseline (V1), months 2, 6, 10, 14, 18, 22, 26. Non-fasted specimens were allowed to clot in 3.5 mL serum separation tubes (ambient temperature, 30–120 min) and then centrifuged within 120 min. Serum analytes were measured in duplicate using Beckman Coulter AU5800 platforms: Cr (Jaffe’s alkaline picrate), CK (IFCC enzymatic assay), and CysC (immunoturbidimetric; all inter-assay CV < 3%). CCR was computed as the creatinine (µmol/L)/cystatin C (mg/L) ratio.

### Functional assessments

A sole evaluator credentialed in SMA-specific metrics performed assessments within 24 h preceding planned nusinersen injections. Testing was conducted at baseline, 2, 14, and 26 months, using validated tools.

HFMSE: Measures gross motor capacity in ambulant/seated SMA subjects aged ≥ 2 years, tracking disease course/treatment impact [25, 26].

RULM: Appraises upper-extremity performance using 19 progressively complex activities (passive motion→purposeful actions) among patients ≥ 30 months possessing sitting ability [12, 27, 28].

HINE-2: Standardized appraisal of infant motor trajectories across eight developmental indicators (cephalic control→unaided ambulation) [13] (Scale particulars: Additional file 1).

### Covariates

The following baseline characteristics were documented: molecular diagnosis age, diagnostic latency (symptom emergence→confirmation), initial nusinersen administration age, gender, and body mass index (BMI, kg/m²). SMN2 copy quantification employed multiplex ligation-dependent probe amplification (SALSA P060). Ambulation capacity (ambulant independently/non-ambulant) and SMA subtype which followed the symptom onset chronology and motor milestone attainment, were assessed [4].

### Ethics approval and consent

The study was approved by the Ethics Committee of the Jinan Children’s Hospital (approval no. SDFE-IRB/T-2025105). Written informed consent was obtained from all parents or legally authorised representatives, specifically permitting the use of de-identified patient data for research purposes. Child assent was obtained when developmentally appropriate.

### Statistical methods

Normality was assessed using the Shapiro-Wilk test. Continuous variables were expressed as median (interquartile range) or mean ± standard deviation. Intergroup baseline contrasts (SMN2 3 copies vs. 4 copies; ambulant vs. non-ambulant) were analyzed using the Mann-Whitney U test with false discovery rate (FDR) correction. We analyzed longitudinal changes in Cr, CK, CysC, and CCR across eight timepoints using Wilcoxon signed-rank tests, with Bonferroni correction applied to account for multiple timepoint comparisons. Linear mixed-effects modelling was used to investigate the biomarker-motor score association (HFMSE/RULM/HINE-2), adjusted for age at treatment initiation, BMI, SMA type, and treatment duration. A logarithmic transformation was applied to all continuous variables to satisfy the model assumptions of linear mixed-effects modeling. Subject-specific random intercepts managed intra-individual dependencies. The magnitudes appear as β coefficients (95% confidence intervals [95% CI] ). Multiple imputations were performed to handle missing data for biomarkers, covariates, and functional scores. The Benjamini-Hochberg procedure was used to control the FDR, and adjusted *p*-values were reported alongside the original *p*-values.

All analyses were performed using R 4.2.2. Linear mixed-effects models (lme4 v1.1.34) employed restricted maximum likelihood estimation, with fixed effects for age, BMI, SMA type, and treatment duration, and random patient intercepts. Model selection compared the Akaike information criterion(AIC) and Bayesian information criteria (BIC). Statistical significance was defined as a two-sided *P* < 0.05.

## Results

### Baseline characteristics

Thirty-three genetically confirmed 5q-SMA paediatric patients completed 264 longitudinal evaluations. The cohort included 14 males and 19 females, with a median age at molecular confirmation of 18 months old. Diagnostic delay varied by subtype: type 2 (7 months, IQR 4.5–15) versus type 3 (18 months, IQR 7.5–29.5), but the differences were not statistically significant(*P* > 0.05). At baseline, eight participants were independently ambulant, and following intrathecal nusinersen initiation, one previously non-ambulant 17-year-old attained an ambulatory capacity. One presymptomatic infant commenced treatment at 40 days postpartum and achieved age-appropriate motor milestone. Subtype distribution: type 1 (*n* = 2), type 2 (*n* = 19), type 3 (*n* = 11), presymptomatic (*n* = 1). The comprehensive baseline characteristics are shown in Table 1.

**Table 1:** Baseline characteristics

Variable	Patients with SMA (*n* = 33)
Sex (male/female)	14/19
BMI (kg/m²)	16.65 ± 4.24
SMA type (1/2/3/presymptomatic)	2/19/11/1
*SMN2 copy number (3/4)	
Number = 3	25
Number = 4	6
Ambulant (yes/no)	8/25
Age of genetic diagnosis(m)	
SMA type 2(*N* = 19)	17.0(13.0,25.0)
SMA type 3(*N* = 11)	35.0(22.5,54.5)
Diagnostic delay (m)	
SMA type 2	7.0(4.5,15)
SMA type 3	18.0(7.5,29.5)
*HFMSE	15(1,61)
**RULM	17(11, 27)
***HINE-2	11.00(9.25, 17.00)
CK (U/L)	116(95,146)
Cr (µmol/L)	8(6,13)
Cystatin C (mg/L)	0.76(0.67,0.83)
CCR(µmol/mg)	11.11(8.49,17.78)

*Abbreviations*: *SMA* spinal muscular atrophy, *BMI* body mass index, *SMN* survival motor neuron, *HFMSE* Hammersmith Functional Motor Scale Expanded (range 0–66), *RULM* Revised Upper Limb Module (range 0–37), *HINE-2* Hammersmith infant neurological Exam-Part 2(range 0–26), *CK (U/L)* serum creatine kinase, *Cr (µmol/L)* serum creatinine, *CCR(µmol/mg)* creatinine-to-cystatin C ratio

*Represents 2 missing

** Represents 12 missing

***Represents 7 missing

### Data completeness

The frequency of missing data for serum biomarkers and functional scores at each assessment time point is summarized in Supplementary Table 2. As shown, functional assessments exhibited substantial missing data, particularly for HINE-2 and RULM. This pattern is largely attributable to the developmental and clinical applicability of these tools; for instance, the RULM is not designed for infants, and the HINE-2 has limited applicability in older children. Missing assessments also frequently occurred due to scheduled visit non-attendance, primarily because of intercurrent illnesses, a common challenge in longitudinal paediatric cohorts with neuromuscular conditions.

### Cross-sectional biomarker profiles

**Fig. 1:**
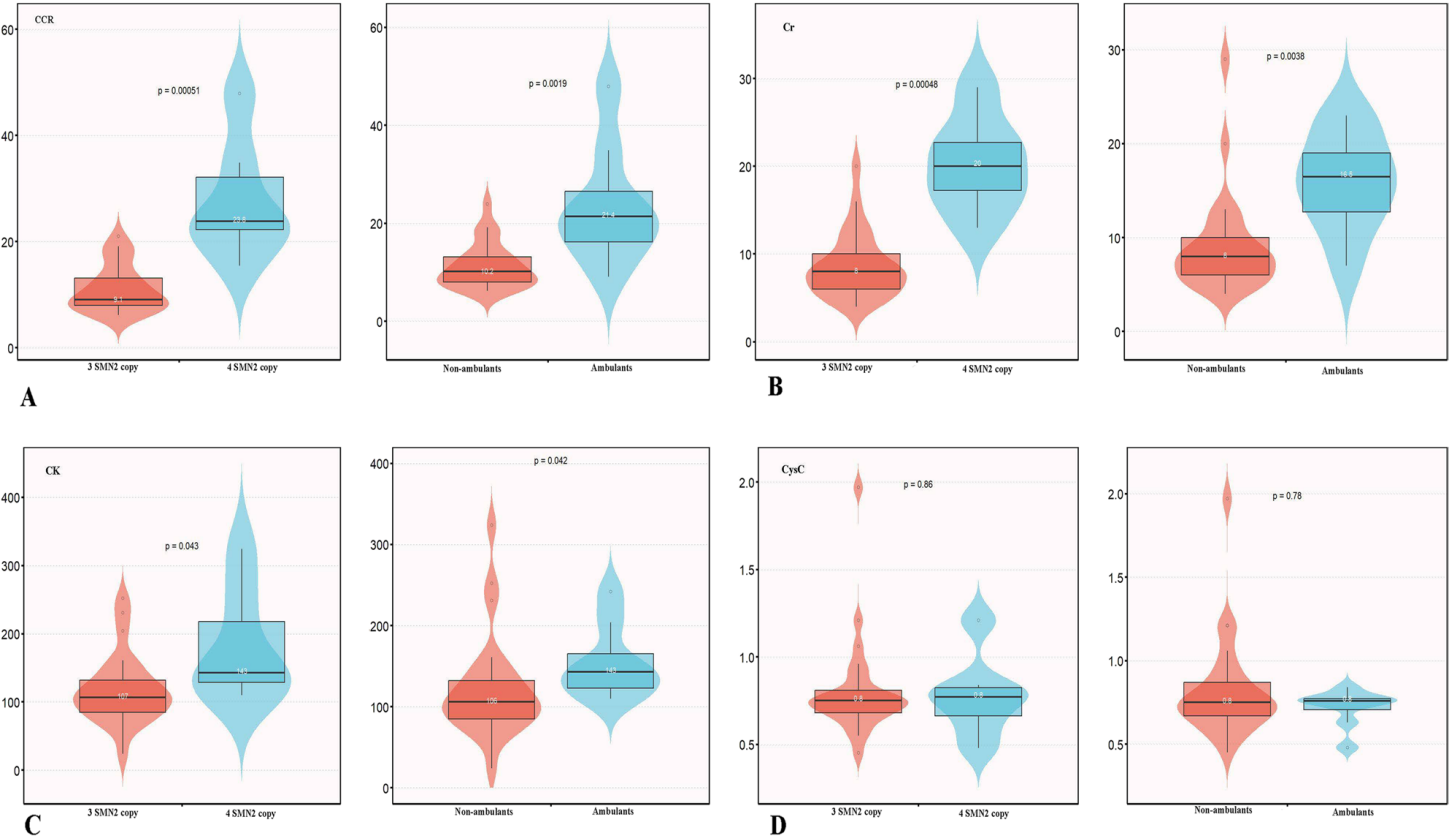
Comparison of serum biomarkers stratified by SMN2 copy number(*n* = 31) and ambulatory status(*n* = 33) at baseline. **A** Creatinine to Cystatin C ratio (CCR); **B** Serum creatinine (Cr); **C** Serum creatine kinase (CK); **D** Cystatin C(CysC). The central line within each box represents the median, and the bounds of the box indicate the interquartile range (IQR). Group comparisons were performed using the Mann-Whitney U test, and FDR correction was applied

Following FDR adjustment, significant differences in CCR and Cr levels were observed across groups stratified by SMN2 copy number and ambulatory status (Fig. 1). Specifically, patients with four SMN2 copies had higher CCR (23.8[22.3, 32.2] vs. 9.1[8.0, 13.2] µmol/mg) and Cr (20.0[17.2, 22.8] vs. 8.0[6.0, 10.0] µmol/L) than those with three copies, and ambulant individuals showed higher levels of these biomarkers than non-ambulant counterparts (CCR: 21.4[16.2, 26.5] vs. 10.2[8.0, 13.2]; Cr:16.5[12.8, 19.0] vs. 8.0[6.0, 10.0]). In contrast, the differences in CK levels were not statistically significant following FDR correction. The serum CysC levels did not differ between the groups.

### Longitudinal biomarker trajectories

**Fig. 2:**
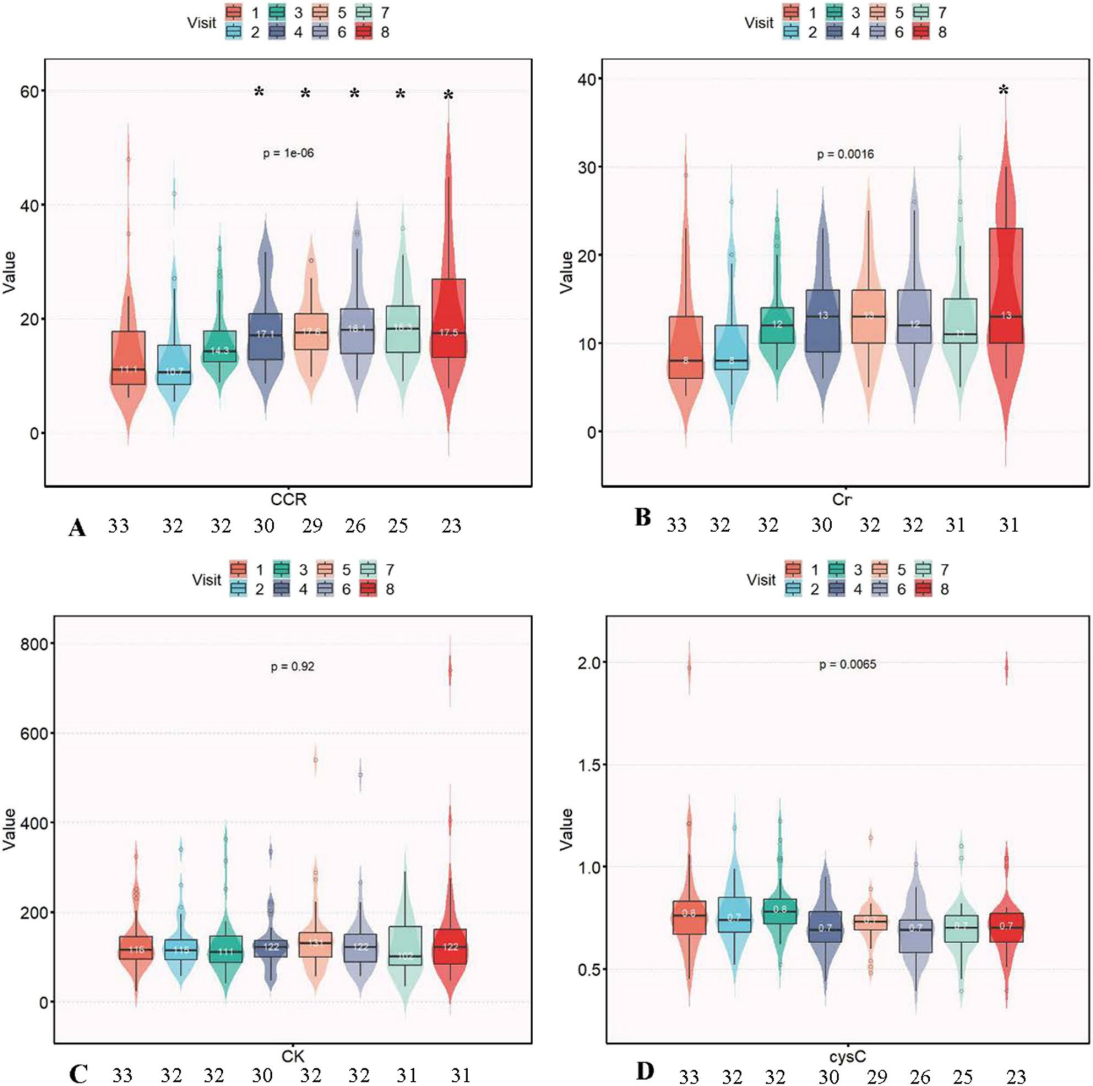
Longitudinal trajectories of serum biomarkers over 26 months of intrathecal nusinersen therapy in 33 children with genetically confirmed 5q-SMA. **A** Creatinine to Cystatin C ratio (CCR); **B** Serum creatinine (Cr); **C** Serum creatine kinase (CK); **D** Cystatin C(Cys C). The number annotated beneath each visit point on the x-axis indicates the actual observed sample size with available data at that time. The solid lines connect the median values at each time point. The asterisk denotes a significant difference from baseline (Month 0), as analyzed using the Wilcoxon signed-rank test. To account for multiple tests across the four follow-up timepoints for each biomarker, the Bonferroni method was applied, setting the significance threshold at *p < 0.007. All 33 patients (*n* = 33) were included at each time point, and missing values were addressed using multiple imputation

The longitudinal trajectories of serum biomarkers throughout 26 months of nusinersen therapy, analyzed following multiple imputations of missing data, are summarized in Fig. 2. CCR showed progressive increases, achieving statistical significance compared to the baseline from visit 4 onwards (all *p* < 0.007). Serum Cr exhibited a modest, gradual elevation, becoming significant only at visit 8 (*p* < 0.007). In contrast, serum CK and CysC levels remained stable over the observation period, with no significant changes from the baseline at any time point (all *p* > 0.007).

### Multivariable motor function associations

In fully adjusted linear mixed-effects models, CCR was associated with higher HFMSE (β = 0.49, 95%CI: 0.20–0.79; *P* = 0.001) and RULM (β = 0.35, 95%CI: 0.11–0.60; *P* = 0.005). The association between CCR and HINE-2 scores was not statistically significant (β = 0.11, 95%CI: -0.06 ~ 0.29; *P* = 0.213). Serum Cr showed positive associations with HFMSE (β = 0.39, 95%CI: 0.06 ~ 0.73; *P* = 0.022) and RULM (β = 0.31, 95%CI: 0.03 ~ 0.58; *P* = 0.029). However, these associations did not retain statistical significance after FDR adjustment. No significant association was observed between Cr and HINE-2 scores. Serum CK levels were inversely associated with RULM scores (β=-0.23, 95%CI: -0.45~-0.01; *P* = 0.046), although this association was not significant after FDR correction. CK showed no significant association with HFMSE or HINE-2 scores. CysC concentrations were significantly inversely correlated with HINE-2 scores (β=-1.14, 95%CI: -1.91~-0.38; *P* = 0.004). Although CysC levels were negatively correlated with HFMSE and RULM scores, these associations were not statistically significant. This analysis was performed using datasets with missing values that were addressed using multiple imputations (Table 2).

**Table 2:** Correlation between biomarkers and functional scores over time (based on multiple imputation analysis and FDR adjustment)

Biomarkers	Motor functional scores	Crude model (β (95% CI))	*P*-value	Adjusted model (β (95% CI))	*P*-value	Adj-*P*(FDR)
LnCCR	LnHFMSE	0.83 (0.58 ~ 1.09)	< 0.001	0.49 (0.2 ~ 0.79)	0.001	**0.012**
	LnRULM	0.63 (0.43 ~ 0.82)	< 0.001	0.35 (0.11 ~ 0.6)	0.005	**0.02**
	LnHINE-2	0.36 (0.2 ~ 0.52)	< 0.001	0.11 (-0.06 ~ 0.29)	0.213	0.284
LnCr	LnHFMSE	0.86 (0.57 ~ 1.15)	< 0.001	0.39 (0.06 ~ 0.73)	0.022	0.066
	LnRULM	0.67 (0.45 ~ 0.89)	< 0.001	0.31 (0.03 ~ 0.58)	0.029	0.07
	LnHINE-2	0.33 (0.15 ~ 0.51)	< 0.001	-0.04 (-0.23 ~ 0.16)	0.706	0.847
LnCK	LnHFMSE	0.22 (-0.11 ~ 0.55)	0.19	-0.02 (-0.29 ~ 0.26)	0.91	0.961
	LnRULM	-0.01 (-0.27 ~ 0.24)	0.92	-0.23 (-0.45~-0.01)	0.046	0.092
	LnHINE-2	0.12 (-0.07 ~ 0.31)	0.226	0 (-0.15 ~ 0.16)	0.961	0.961
LgCystatin C	LnHFMSE	-1.72 (-3.26~-0.17)	0.031	-1.28 (-2.65 ~ 0.09)	0.069	0.118
	LnRULM	-1.34 (-2.51~-0.17)	0.027	-0.92 (-2.02 ~ 0.18)	0.105	0.158
	LnHINE-2	-1.34 (-2.21~-0.46)	0.003	-1.14 (-1.91~-0.38)	0.004	**0.02**

*Abbreviations*: *CK(U/L)* creatine kinase, *Cr (µmol/L)* creatinine, *CCR(µmol/mg)* creatinine-to-cystatin C ratio, *HFMSE* Hammersmith Functional Motor Scale Expanded, *RULM* Revised Upper Limb Module, *HINE-2* Hammersmith infant neurological Exam-Part 2, *FDR* False Discovery Rate, *CI* Confidence Interval

Significant based on the linear mixed-effects model with baseline age, body mass index, treatment duration, and SMA type as covariates; bold FDR-adjusted *p*-values indicate significance at adj-*p* < 0.05. Missing values were addressed using multiple imputation. All biomarkers and functional scores were transformed using logarithmic transformation

## Discussion

This study established serum Cr dynamics as a phenotypic indicator of SMA. Reduced Cr concentrations in patients with three SMN2 copies and non-ambulant individuals at baseline confirmed their association with disease severity. Serum Cr showed positive but non-significant associations with motor scores after FDR adjustment. Our findings extend the established neuromuscular evidence that diminished Cr consistently reflects advanced pathology in spinal and bulbar muscular atrophy [29, 30] and dystrophinopathies [31, 32]. Alves et al. [17] first documented paediatric Cr reductions in severe SMA (0–55.2 months), which were subsequently validated in adult cohorts, where Cr demonstrated prognostic value [15, 33–35]. The temporal evolution of Cr levels clarifies their role in clinical practice. Initial nusinersen therapy (≤ 2 months) induces functional improvement without altering Cr levels [36]. Our longitudinal data revealed elevated Cr levels 26 months after the baseline. This delayed response may reflect chronic neuromuscular adaptation, suggesting Cr as a marker of sustained neuromuscular status rather than acute improvement. However, these interpretations remain hypothetical and require validation through electrophysiological, imaging, or histopathological correlates to establish the underlying mechanisms of this phenomenon. Furthermore, the utility of serum Cr levels for monitoring neuromuscular status in SMA may be influenced by confounding factors. These include: (1) potential renal function alterations from SMA pathology or therapies [37] may confound interpretation and (2) extramuscular factors (diet and exercise) may obscure the Cr-muscle relationships. Therefore, future studies should control for these variables to optimize their utility.

As a muscle mass-independent renal marker, CysC stability throughout 26 months of nusinersen therapy confirmed renal safety in our cohort, consistent with previous SMA reports [38, 39]. Beyond the renal context, emerging evidence implicates CysC in neural injuries. In Parkinson’s disease, elevated CysC levels correlate with neurofilament light chain levels, and longitudinal data link higher baseline levels to accelerated motor decline [40]. Our observation of an inverse correlation between CysC levels and motor function scores in SMA aligns with this emerging concept of CysC as a potential biomarker associated with neural dysfunction. Although SMA primarily affects motor neurons, the elevated CysC levels observed in our patients may indicate the presence of secondary neurodegenerative processes or potentially compromised renal clearance. These factors could contribute to or be associated with the amplification of neural damage signals. Specifically, the negative association between CysC and the HINE-2 score is a novel finding that has not been previously reported in the SMA literature. While we adjusted for clinical covariates, residual confounding by unmeasured factors (e.g. renal tubular function, medications affecting CysC metabolism, and undetected comorbidities) cannot be excluded. The clinical relevance of this association remains speculative and requires further mechanistic investigations to elucidate causality.

Our data demonstrated that a reduced CCR was associated with greater disease severity, and its elevation was detectable 10 months after initiation, underscoring its enhanced responsiveness. In addition, CCR levels were positively correlated with the motor function scores. By mathematically incorporating CysC to estimate GFR, CCR mitigates renal confounding, a critical advancement that addresses the limitations of renal comorbidities [37, 41]. Notably, CCR was selected as a biomarker due to its established correlation with muscle mass in SMA and reduced susceptibility to acute fluctuations compared to Cr alone. However, its renal dependence necessitates nuanced interpretation, particularly in the paediatric population, where GFR maturation varies nonlinearly with age. This innovation establishes CCR as a more reliable lean mass surrogate in populations with varying renal functions. Converging evidence supports the validity of the CCR: studies show that in adults with SMA, CCR dynamically correlates with HFMSE scores during nusinersen therapy [14]; it correlates with skeletal muscle mass in kidney transplant recipients [42] and in chronic kidney disease patients with renal-muscle axis impairment [43], and it demonstrates diagnostic power for sarcopenia detection and mortality prediction [23]. Most compellingly, Tetsuka et al. confirmed the utility of the CCR in quantifying residual muscle mass in amyotrophic lateral sclerosis [44], directly supporting the applicability of neurogenic atrophy.

Initial analysis suggested elevated CK levels in ambulant patients and those with four SMN2 copies, although statistical significance was lost after correction. Although a negative CK-motor function trend was observed, our longitudinal data confirmed no significant CK changes over 26 months. These findings contrast with some adult SMA studies, such as Freigang et al., who reported declining CK levels alongside clinical improvement [15], and a recent Chinese cohort that observed inverse baseline correlations but reported no longitudinal shifts [14]. Based on our findings, the mechanism proposed in adults, where superior baseline motor function (potentially reflecting greater muscle mass and susceptibility to subclinical injury) predicts CK elevation, may not fully extend to paediatric SMA cases. The consistent lack of significant longitudinal changes in CK levels in children suggests that CK is not a reliable biomarker for tracking disease progression in paediatric SMA. Potential reasons for this discrepancy between paediatric and adult findings include the masking of muscle-specific signals by common biological confounders (e.g. exertion, minor injury, and medication) [45], potential differences in paediatric neuromuscular biology affecting turnover and repair kinetics, and the absence of standardized normalization approaches (e.g. lean mass), which may introduce heterogeneity. In summary, although CK levels may exhibit phenotypic associations at baseline, our data indicate that they are not a reliable biomarker for tracking disease progression in paediatric SMA.

Following the initial loading phase of nusinersen therapy, the dosing regimen transitions to maintenance administration every four months. Synchronizing the measurement of the CCR with these routine treatment visits (i.e., at each infusion) represents a highly practical and operationally feasible approach. This strategy minimizes the additional burden on patients and leverages existing clinical touchpoints, thereby facilitating seamless integration into standard monitoring protocols. The delayed emergence of significant CCR elevation (approximately 10 months) further underscores the necessity of longitudinal assessment beyond the initial treatment phase to capture the dynamic changes in biomarkers reflecting neuromuscular adaptation. Through Receiver Operating Characteristic analysis, we identified two exploratory CCR thresholds: 3.59 µmol/mg (associated with clinically meaningful changes in HFMSE) and 7.94 µmol/mg (associated with clinically meaningful changes in RULM) (Additional file 2). These exploratory thresholds demonstrate preliminary utility for hypothesis generation but exhibit limited predictive accuracy (AUC 0.63–75) in clinical decision-making. Future studies should refine these thresholds using larger cohorts integrated with clinical outcome data to improve their discriminative ability. The CCR offers distinct practical advantages for routine clinical use. Unlike complex functional assessments, such as the HFMSE and RULM, which require specialized training, are time-consuming, and may exhibit floor or ceiling effects [10–13], CCR is derived from routine, minimally invasive blood draws that measure serum Cr and CysC. These assays are widely available, standardized, and inexpensive. Coupled with its straightforward calculation (Cr / Cys C), CCR has emerged as a highly accessible and practical biomarker in diverse global healthcare settings.

### Strengths and limitations

This study benefited from longitudinal biomarker assessments conducted over ≥ 26 months, employing frequent serial measurements to enhance temporal resolution. Methodological rigor was maintained through stringent assay standardization protocols and blinded physiotherapist assessments of functional outcomes. However, this study has several limitations. 1) A key limitation is the substantial proportion of missing motor function assessments, particularly in later visits (Supplementary Table 1). Although multiple imputation under the Missing at Random (MAR) assumption was applied, supported by sensitivity analyses showing consistent results, the high degree of missingness may affect the stability of mixed-effects model estimates. Specifically, it can reduce the precision of subject-specific random effects (e.g. intercepts and slopes) and introduce uncertainty in estimating true longitudinal trajectories, especially during critical post-treatment periods. Furthermore, if missingness is related to unobserved outcomes (i.e. missing not at random, MNAR), residual bias could persist despite imputation. While we aimed to minimize this risk by incorporating a rich set of covariates into the imputation model, the potential impact of missing data on the stability and potential bias of the estimates remains a notable limitation.2) The interpretation of CCR in our paediatric cohort is inherently confounded by renal development. The absence of direct renal function parameters (e.g. eGFR, BUN, urinalysis) precludes the definitive disentanglement of renal versus neuromuscular contributions to CCR dynamics. Although CysC exhibits greater stability than Cr in cases of renal impairment, CCR remains partially dependent on GFR. Consequently, we cannot fully rule out renal confounding or attribute CCR changes exclusively to neuromuscular adaptation or treatment response in this study. Future studies incorporating direct renal function assessments are warranted to validate CCR’s utility of CCR in paediatric SMA. 3) Third, although CCR serves as a functional surrogate for muscle mass in neuromuscular disorders, the absence of direct anatomical measurements (e.g. dual-energy X-ray absorptiometry [DXA], quantitative MRI, or ultrasound) in this cohort limits our ability to biologically validate CCR as a direct correlate of muscle mass. Future studies should prioritize the integration of CCR measurements with quantitative muscle imaging modalities to validate their biological accuracy as measures of muscle mass. 4) Fourth, the derived ΔCCR thresholds, while statistically significant, demonstrated only a modest discriminatory capacity (AUC 0.63–0.75). This implies limited standalone clinical applicability, and their utility should be validated prospectively, alongside established functional endpoints. 5) Others: Recruitment from a single institution introduces potential selection bias, thereby limiting the generalisability of the results. While our analyses demonstrated statistically significant associations between biomarker dynamics and functional outcomes, the observational design of the study inherently precludes definitive conclusions regarding causality. Although key covariates were adjusted for in the mixed-effects models, the possibility of residual confounding influencing the relationship between biomarker changes and motor function improvement cannot be fully excluded. Subsequent validation efforts should prioritize longitudinal multicenter verifications in larger cohorts.

## Conclusion

CCR is a potential prognostic biomarker in paediatric patients with SMA receiving nusinersen monotherapy. However, its validity and generalizability require confirmation through prospective multicenter studies involving larger and more diverse patient cohorts. Because CCR is a simple, inexpensive, and accessible measurement, we propose that CCR should be routinely monitored in the longitudinal follow-up of all patients with SMA.

## Supplementary Information

Supplementary Material 1.

Supplementary Material 2.

Supplementary Material 3.

Supplementary Material 4.

## Data Availability

The datasets used and/or analyzed during the current study are available fromcorresponding author upon reasonable request.

## Abbreviations

SMA: Spinal Muscular Atrophy
CCR: Creatinine-to-cystatin C Ratio
Cr: Creatinine
CK: Creatine Kinase
HFMSE: Hammersmith Functional Motor Scale-Expanded
RULM: Revised Upper Limb Module
HINE-2: Hammersmith infant neurological Exam-Part 2
SMN 1: Survival Motor Neuron 1
DMTs: Disease-Modifying Therapies
GFR: Glomerular Filtration Rate
CysC: Cystatin C
DXA: Dual-Energy X-ray Absorptiometry
IQR: Interquartile Range
SD: Standard Deviation
CI: Confidence Interval
BMI: Body Mass Index
