# Supplementary material for: Serum creatinine to cystatin C ratio as a biomarker for monitoring motor-function in children with spinal muscular atrophy treated with nusinersen: a retrospective cohort study
Source: BMC Neurol. 2026 Jan 24;26:120. doi: 10.1186/s12883-026-04657-3 (PMC12910726; doi:10.1186/s12883-026-04657-3)
Supplement: Supplementary file 1 — Supplementary Material 1. [file 12883_2026_4657_MOESM1_ESM.docx]

1. Specific content of the Expanded Hammersmith Functional Motor Scale for SMA (HFMSE)(1)

| Test item | Instruction | 2 | 1 | 0 |
| --- | --- | --- | --- | --- |
| 1.Plinth/chair sitting | Can you sit on the plinth/chair without using your hands for support for a count of 3? | Able to sit using no hand | Needs one hand support to | Needs two hand support to |
|  |  | support for a count of 3 or | maintain balance for a count | maintain balance |
|  |  | more | of 3 | Unable to sit |
| 2.Plinth /chair sitting | Can you sit on the floor/plinth without using your hands for support and with your legs straight | Able to sit on floor/plinth | Able to sit on floor/plinth | Able to long sit using two |
|  | for a count of 3? Don’t let your legs roll out. | with legs straight without | with legs straight propping | hands for a count of 3 |
|  |  | hand support for a count of 3 | with one hand support for a | Or unable to sit with straight |
|  |  |  | count of 3 | legs |
| 3.One hand to head in sitting | Can you get one hand to your head (above your ear) without bending your neck? | Able to bring one hand to | Can only bring hand to head | Unable to bring hand to |
|  |  | head. Head and trunk | by flexing head | head even using head and |
|  |  | remain stable |  | trunk movement |
| 4.Two hands to head in sitting | Can you lift both hands up at the same time, to your head, without bending your neck? | Able to place both hands on | Able to place hands on head | Unable to place both hands |
|  |  | head arms free from side. | but only using head flexion | on head |
|  |  | Head and trunk remain | or side tilt or crawling hands |  |
|  |  | stable | up or one at a time |  |
| 5.Supine to side lying | Can you roll onto your side in both directions? | Able to ½ roll from supine | Can ½ roll only one way | Unable to half roll either way |
|  |  | both ways | R / L |  |
| 6.Rolls prone to supine over R | Can you roll from your tummy to your back in both directions? Try not to use your hands | Turns to supine with free | Turns to supine using arms | Unable to turn to supine |
|  |  | arms to the right | to push / pull with |  |
| 7.Rolls prone to supine over L | Can you roll from your tummy to your back in both directions? Try not to use your hands | Turns to supine with free | Turns to supine using arms | Unable to turn to supine |
|  |  | arms to the left | to push / pull with |  |
| 8.Rolls supine to prone over R | Can you roll from your back to your tummy in both directions? Try not to use your hands | Turns to prone with free | Turns to prone by pulling | Unable to turn into prone |
|  |  | arms to the right | /pushing on arms |  |
| 9.Rolls supine to prone over L | Can you roll from your back to your tummy in both directions? Try not to use your hands | Turns to prone with free | Turns to prone by pulling / | Unable to turn into prone |
|  |  | arms to the left | pushing on arms |  |
| 10.Sitting to lying | Can you lie down in a controlled way from sitting? | Able to lie down in a | Able to lie down by flopping | Unable or falls over |
|  |  | controlled fashion through | forwards and rolling |  |
|  |  | side lying or using clothes | sideways |  |
| 11.Props on forearms | Can you prop yourself on your forearms and hold for a count of 3? | Able to achieve prop on | Holds position for a count of | Unable |
|  |  | forearms with head up for | 3 when placed |  |
|  |  | count of 3 |  |  |
| 12.Lifts head from prone | Can you lift your head up keeping your arms by your side for a count of 3? | Able to lift head up in prone | Lifts head with arms in a | Unable |
|  |  | arms by side for a count of 3 | forward position for a count |  |
|  |  |  | of 3 |  |
| 13.Prop on extended arms | Can you prop yourself up with straight arms for a count of 3? | Able to prop on extended | Can prop on extended arms | Unable |
|  |  | arms, head up for a count of | if placed for a count of 3 |  |
|  |  | 3 |  |  |
| 14.Lying to sitting | Can you get from lying to sitting without rolling to your tummy? | Able by using side lying | Turns into prone or towards | Unable |
|  |  |  | floor |  |
| 15.Four–point kneeling | Can you get onto your hands and knees with your head up and hold for a count of 3? | Achieves four-point | Holds position when placed | Unable |
|  |  | kneeling. Head up for a | for a count of 3 |  |
|  |  | count of 3 |  |  |
| 16.Crawling | Can you crawl forwards? | Able to crawl forwards | Moves all four points only | Unable |
|  |  | Moves all four points twice | once |  |
|  |  | or more |  |  |
| 17.Lifts head from supine | Can you lift your head to look at your toes keeping your arms folded for a count of 3 | In supine, head must be | Head is lifted but through | Unable |
|  |  | lifted in mid-line. Chin | side flexion or with no neck |  |
|  |  | moves towards chest. Held | flexion. Held for a count of 3. |  |
|  |  | for a count of 3. |  |  |
| 18.Supported standing | Can you stand using one hand for support for a count of 3? | Can stand with one hand | Able to stand with minimal | Unable |
|  |  | support for a count of 3. | trunk support (not hip) for a |  |
|  |  |  | count of 3. |  |
| 19.Stand unsupported | Can you stand without holding onto anything for a count of 3? | Can you stand without holding onto anything for a count of 3? | Stands independently for a | Stands only momentarily |
|  |  |  | count of 3 | (less than a count of 3) |
|  |  |  |  | Or unable |
| 20.Stepping | Can you walk without using any help or aids? Show me | Able to take more than 4 | Able to take 2 – 4 steps | Unable |
|  |  | steps unaided | unaided |  |
| 21.Right hip flexion in supine | Can you bring your right knee to your chest? | Full hip flexion achieved | Full hip flexion achieved | Unable |
|  | Individuals should not use their arms to assist this activity |  |  |  |
| 22.Left hip flexion in supine | Can you bring your left knee to your chest? | Full hip flexion achieved | Initiates left hip and knee | Unable |
|  | Individuals should not use their arms to assist this activity |  | flexion |  |
|  |  |  | (more than 10% of available |  |
|  |  |  | range of motion) |  |
| 23.High kneeling to right half kneel | Can you bring your left leg up so that your foot is flat on the ground without using your arms | The subject transitions from high kneeling to half kneeling on right knee, with or | Maintains half kneel with | Unable |
|  | and hold for a count of 10? | without the use of arm support, and then maintains right half kneel for | arm support |  |
|  |  | without arm support. |  |  |
| 24.High kneeling to left half kneel | Can you bring your right leg up so that your foot is flat on the ground without using your arms | Arms used for transition, | Maintains half kneel with | Unable |
|  | and hold for a count of 10? | maintains arms free for half | arm support |  |
|  |  | kneel |  |  |
| 25.High kneeling to standing, leading with left leg (through right half kneel) | Can you stand up from this position starting with your left leg without using your hands? | Able with arms free | Able to shift weight off both | Unable |
|  |  |  | knees (with or without arm |  |
|  |  |  | support) |  |
| 26.High kneeling to standing leading with right leg (through left half kneel) | Can you stand up from this position starting with your right leg without using your hands? | Able with arms free | Able to shift weight off both | Unable |
|  |  |  | knees (with or without arm |  |
|  |  |  | support) |  |
| 27.Stand to sitting on the floor | Can you sit on the floor,in a controlled way?Try not to use your arms. | Able to sit down with arms | Sits on floor but uses arms or | Unable |
|  | Sitting can be any style of comfort,i.e.short sitting,long sitting,frog sitting | free and no collapse | crashes |  |
| 28.Squat | Can you squat? Pretend you are going to sit in a very low seat | Squats with arms free | Initiates squat (more than | Unable to initiate |
|  |  |  | 10%) , uses arm support |  |
| 29.Jumps 12 inches forward | Can you jump as far as you can, with both feet, from this line all of the way to the other line? | Jumps at least 12”, both feet | Jumps between 2- 11”, both | Unable to initiate jump with |
|  |  | simultaneously | feet simultaneously | both feet simultaneously |
| 30.Ascends 4 stairs with railing | Can you walk up the steps? You can use one railing | Ascends 4 stairs with railing, | Ascends 2-4 stairs, one rail, | Unable to ascend 2 stairs using one rail |
|  |  | alternating feet | any pattern |  |
| 31.Descends 4 stairs with railing | Can you walk down the steps? You can use one railing | Descends four stairs , with | Descends 2-4 stairs, one rail, | Unable to descend 2 stairs |
|  |  | railing, alternating feet | any pattern | with one rail |
| 32.Ascends 4 stairs without arm support | Can you walk up the steps? This time try not to use the railing | Ascends four stairs, arms | Ascends 2-4 stairs, arms | Unable to ascend 2 stairs |
|  |  | free, alternating feet | free, any pattern | arms free |
| 33.Descends 4 stairs without arm support | Can you walk down the steps? This time try not to use the railing. | Descends four stairs, arms | Descends 2-4 stairs, arms | Unable to descend 2 stairs |
|  |  | free, alternating feet | free, any pattern | arms free |

2. Specific content of the Revised Upper Limb Module for SMA(RULM) scale (2).

| Items | 0 | 1 | | 2 | 3 | | 4 | | 5 | | 6 |
| --- | --- | --- | --- | --- | --- | --- | --- | --- | --- | --- | --- |
| A.1.Lift your hands and arms as high as you can.2. Can you bring your hands to your  mouth? | No useful  function of  hands. | Can use hands  to hold pencil  or pick up a  token or drive a  powered chair,  use phone key  pad | | Can raise 1 or 2  hands to  mouth but  cannot raise a  cup with a 200g  weight in it to  mouth | Can raise  standardized  plastic cup with  200g weight in  it to mouth  using both  hands if  necessary. | | Can raise both  arms  simultaneously  to shoulder  height with or  without  compensation.  Elbow bent or  in extension | | Can raise both  arms  simultaneously  above head  only by flexing  the elbow  shortening  circumference  of the  movement  /using  accessory  muscles) | | Can abduct  both arms  simultaneously  elbows in  extension in a  full circle until  they touch  above the  head. |
| Items | | | Instruction | | | 0 | | 1 | | 2 | |
| B. Bring Hands from lap to table | | | Bring both hands from lap to table at the same time. | | | Unable | | One hand completely on  table | | Two hands completely and  simultaneously on table or  one at the time | |
| C. Tracing a path | | | Can you complete the path bringing the car to the finish line  without stopping or taking pencil off of paper? | | | Unable | | Completes path but stops  or raises pencil along the  path | | Able to complete path  without stopping or  raising hand | |
| D. Picking up coins / tokens | | | Can you pick up these coins one at a time with one hand and  hold them? | | | Unable | | Picks up and holds 1  coin in one hand | | Picks up and holds 2  coins in one hand | |
| E. Place token into cup on table or at shoulder height | | | Can you place the token in the cup without throwing ?” – any  strategy | | | Unable | | Able to place token in  cup lying horizontally | | Able to place token in  cup placed vertically at  shoulder height | |
| F. Reach to the side and touch token | | | Token placed at 90 degrees  shoulder abduction and wrist length  from patient. If patient succeeds  then place token at 135 degrees  shoulder abduction at fingertip  length from patient | | | Unable | | Target: 90° shoulder abduction, token  at wrist length. Brings hand to  shoulder height, elbow flexed or  extended | | Target: 135° shoulder abduction, token  at fingertip length. Brings hand above  shoulder height, elbow at least at eye  level | |
| G. Push on light | | | Can you turn the light on by pushing it, hard enough to keep it  lit? Try using one hand first. | | | Unable with one hand | | Able to turn on light momentarily  with fingers of and/or thumb of  one hand | | Able to turn on light permanently  with fingers and/or thumb of one  hand | |
| H. Tearing paper | | | Can you tear this paper starting from the folded edge? | | | Unable | | Tears sheet of paper  folded in half | | Tears sheet of paper  folded in four | |
| I. Remove lid from container | | | Can you open and take the lid from this container? | | |  | | Opens completely, on  table or against body | | Unable to open | |
| J. Raise cup with 200g to mouth | | | Can you raise the cup to your mouth as if you were drinking? | | | Unable | | Brings 200g in cup to  mouth with two hands | | Brings 200g in cup to  mouth with one hand | |
| K. Move 200g weight on table horizontally | | | Can you lift this weight from the center circle to the outside  circle with one hand? | | | Unable with one  hand | | Slides weight | | Lifts weight | |
| L. Move 500g weight on table horizontally | | | Can you lift this weight from the center circle to the outside  circle with one hand? | | | Unable with one  hand | | Slides weight | | Lifts weight | |
| M. Move 200g weight on table diagonally | | | Can you move this weight from the center circle to this far one  with one hand? | | | Unable with one  hand | | Slides weight | | Lifts weight | |
| N. Raise 500g sand weight from lap to table | | | Hold your hands to receive  weight at patient eye level  and at wrist distance | | | Unable | | Brings weight onto table  using two hands | | Brings weight to eye level  using two hands | |
| O. Repeat - do not take score from Entry item | | | Bring both hands above head | | | Unable | | Can raise both arms simultaneously  above head only by flexing the elbow (with  compensation) – end position must be full  available extension | | Can abduct both arms  simultaneously elbows in  extension in a full circle until they touch above  the head / patient elbows close to ears | |
| P. BRING 500G ABOVE SHOULDER HEIGHT (ABD) | | | Can you give me the weight?” | | | Unable | | Able with  compensation | | Able without  compensation | |
| Q. BRING 1KG ABOVE SHOULDER HEIGHT (ABD) | | | Can you give me the weight?” | | | Unable | | Able with  compensation | | Able without  compensation | |
| R. BRING hand ABOVE SHOULDER HEIGHT (flex) | | | Can you give me the weight? | | | Unable | | Able with  compensation | | Able without  compensation | |
| S. BRING 500G ABOVE SHOULDER HEIGHT (flex) | | | Can you give me the weight? | | | Unable | | Able with  compensation | | Able without  compensation | |
| T. BRING 1kG ABOVE SHOULDER HEIGHT (flex) | | | Can you give me the weight? | | | Unable | | Able with  compensation | | Able without  compensation | |

3. Specific content of the Hammersmith Infant Neurological Exam-Part 2(HINE-2) scale (3).

| Items/score | 0 | 1 | 2 | 3 | 4 |
| --- | --- | --- | --- | --- | --- |
| Head Control | Unable to maintain upright | Wobbles | Maintain upright |  |  |
| Sitting | Cannot sit | Sit with Support at hips | Props | Stable sit | Pivots |
| Voluntary Grasp | No grasp | Uses whole hand | Index finger& thumb | Pincer grasp |  |
| Ability to Kick | No kick | Kick horizontal | Upward | touches leg | Touches toes |
| Rolling | No rolling | Roll to side | Prone to supine | Supine to prone |  |
| Crawling | Does not lift head | On elbow | On outstretched hand | On abdomen | On hands and knees |
| Standing | Not support weight | Support weight | Stands with support | Stands unaided |  |
| Walking | No walking | Bouncing | Cruising | Walk independently |  |

**References**

1. Jennifer Trust for Spinal Muscular Atrophy. (2009, March 7). Expanded Hammersmith Functional Motor Scale for SMA (HFMSE). PNCR Network for SMA.
2. Mayhew, A. (2022, June 20). Revised Upper Limb Module for SMA (RULM). OpenTact.
3. Bishop KM, Montes J, Finkel RS. Motor milestone assessment of infants with spinal muscular atrophy using the hammersmith infant neurological Exam-Part 2: Experience from a nusinersen clinical study. *Muscle Nerve*. 2018;57(1):142-146. doi:10.1002/mus.25705IF: 3.4 Q2
