# Supplementary material for: Serum creatinine to cystatin C ratio as a biomarker for monitoring motor-function in children with spinal muscular atrophy treated with nusinersen: a retrospective cohort study
Source: BMC Neurol. 2026 Jan 24;26:120. doi: 10.1186/s12883-026-04657-3 (PMC12910726; doi:10.1186/s12883-026-04657-3)
Supplement: Supplementary file 2 — Supplementary Material 2. [file 12883_2026_4657_MOESM2_ESM.docx]

**Table 1** Optimal CCR change thresholds for predicting clinically meaningful functional improvements.

|  | Optimal Threshold | Sensitivity | Specificity | AUC | AUC_CI_Lower | AUC_CI_Upper |
| --- | --- | --- | --- | --- | --- | --- |
| HFMSE improvement | 3.59 | 0.75 | 0.54 | 0.63 | 0.43 | 0.84 |
| RULM improvement | 7.94 | 0.56 | 0.88 | 0.75 | 0.58 | 0.92 |

Abbreviations: CCR, creatinine-to-cystatin C ratio; HFMSE, Hammersmith Functional Motor Scale Expanded; RULM, Revised Upper Limb Module; AUC, area under the curve; CI, confidence interval. Optimal thresholds were determined using Youden's index, which represents the best cutoff points for CCR changes in predicting clinically meaningful improvements. Sensitivity reflects the ability to correctly identify true positives (those with clinical improvement), whereas specificity reflects the ability to correctly identify true negatives (those without clinical improvement). AUC values of: 0.5-0.7 indicate limited predictive value, 0.7-0.9 indicate moderate predictive value, and >0.9 indicate high predictive value.


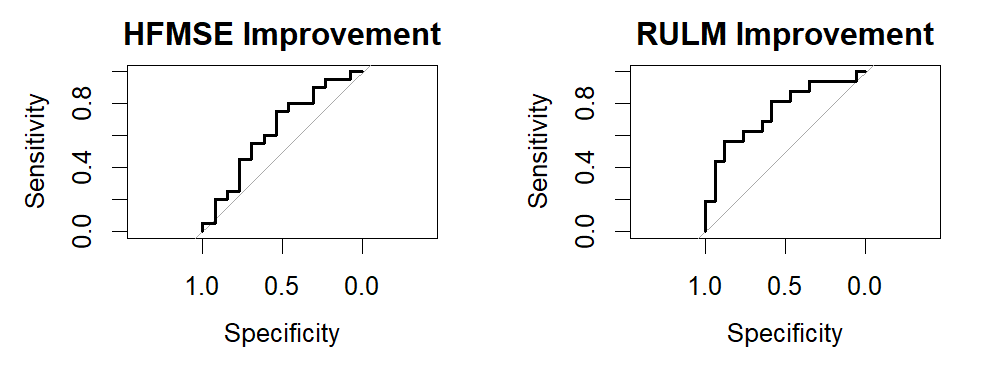


**Figure 1.** ROC analysis of CCR changes predicting functional improvements in SMA. (A)HFMSE improvement: ΔCCR ≥3.59 units (AUC=0.63). (B) RULM improvement: ΔCCR ≥7.94 units (AUC=0.75). The dashed line indicates chance discrimination (AUC=0.5). Solid markers denote optimal thresholds (Youden’s index).

**Abbreviations**: ROC, Receiver Operating Characteristic; CCR, creatinine-to-cystatin C ratio; HFMSE, Hammersmith Functional Motor Scale Expanded; RULM, Revised Upper Limb Module; AUC, area under the curve.
