# Supplementary material for: Serum creatinine to cystatin C ratio as a biomarker for monitoring motor-function in children with spinal muscular atrophy treated with nusinersen: a retrospective cohort study
Source: BMC Neurol. 2026 Jan 24;26:120. doi: 10.1186/s12883-026-04657-3 (PMC12910726; doi:10.1186/s12883-026-04657-3)
Supplement: Supplementary file 3 — Supplementary Material 3. [file 12883_2026_4657_MOESM3_ESM.docx]

| **Correlation between biomarkers and HFMSE scores.** | | | | |
| --- | --- | --- | --- | --- |
| **Variable** | Crude model | | Adjusted model | |
|  | AIC | BIC | AIC | BIC |
| CCR | 764.286 | 774.543 | 714.414 | 724.501 |
| Cr | 760.862 | 771.12 | 715.146 | 725.233 |
| CK | 820.84 | 831.098 | 749.205 | 759.292 |
| CysC | 818.777 | 829.035 | 733.844 | 743.932 |

**Supplementary Table 1** An overview of the correlation analysis, with the relative fit of each model indicated by AIC and BIC values.

| **Correlation between biomarkers and RULM scores.** | | | | |
| --- | --- | --- | --- | --- |
| **Variable** | Crude model | | Adjusted model | |
|  | AIC | BIC | AIC | BIC |
| CCR | 525.672 | 534.888 | 502.605 | 511.599 |
| Cr | 515.968 | 525.184 | 499.016 | 508.01 |
| CK | 552.442 | 561.658 | 520.969 | 529.963 |
| CysC | 543.231 | 552.447 | 508.189 | 517.183 |

| **Correlation between biomarkers and HINE-2 scores.** | | | | |
| --- | --- | --- | --- | --- |
| **Variable** | Crude model | | Adjusted model | |
|  | AIC | BIC | AIC | BIC |
| CCR | 225.83 | 232.051 | 208.088 | 213.824 |
| Cr | 222.313 | 228.534 | 206.759 | 212.495 |
| CK | 254.885 | 261.106 | 214.327 | 220.063 |
| CysC | 247.89 | 254.112 | 203.414 | 209.15 |

Abbreviations: CK, serum creatine kinase; Cr, serum creatinine; CCR, creatinine-to-cystatin C ratio; HFMSE, Hammersmith Functional Motor Scale Expanded; RULM, Revised Upper Limb Module; HINE-2, Hammersmith infant neurological Exam-Part 2; AIC, Akaike Information Criterion; BIC, Bayesian Information Criterion.
