# Supplementary material for: Serum creatinine to cystatin C ratio as a biomarker for monitoring motor-function in children with spinal muscular atrophy treated with nusinersen: a retrospective cohort study
Source: BMC Neurol. 2026 Jan 24;26:120. doi: 10.1186/s12883-026-04657-3 (PMC12910726; doi:10.1186/s12883-026-04657-3)
Supplement: Supplementary file 4 — Supplementary Material 4. [file 12883_2026_4657_MOESM4_ESM.docx]

**Supplementary Table 2** Number of missing values for each variable by visit.

| Missing/n(%) | Visit 1 | Visit 2 | Visit 3 | Visit 4 | Visit 5 | Visit 6 | Visit 7 | Visit 8 |
| --- | --- | --- | --- | --- | --- | --- | --- | --- |
| CCR | 0 | 1(3) | 1(3) | 3(9) | 4(12.1) | 7(21.2) | 8(24.2) | 10(30.3) |
| CK | 0 | 1(3) | 1(3) | 3(9) | 2(6) | 2(6) | 3(9) | 2(6) |
| Cr | 0 | 1(3) | 1(3) | 3(9) | 1(3) | 1(3) | 2(6) | 2(6) |
| Cystatin C | 0 | 1(3) | 1(3) | 3(9) | 4(12.1) | 7(21.2) | 8(24.2) | 10(30.3) |
| HFMSE | 2(6) | 8(24.2) |  |  | 4(12.1) |  |  | 11(33.3) |
| RULM | 12(36.4) | 14(42.4) |  |  | 11(33.3) |  |  | 15(45.5) |
| HINE-2 | 7(21.2) | 18(54.5) |  |  | 16(48.5) |  |  | 18(54.5) |

**Abbreviations**: CK(U/L), creatine kinase; Cr (umol/L), creatinine; CCR(umol/mg), creatinine-to-cystatin C ratio; HFMSE, Hammersmith Functional Motor Scale Expanded; RULM, Revised Upper Limb Module; HINE-2, Hammersmith infant neurological Exam-Part 2.
